# Stable in a Genome of Instability: An Interview with Evan Eichler

**DOI:** 10.1371/journal.pgen.1000124

**Published:** 2008-07-25

**Authors:** Jane Gitschier

**Affiliations:** Departments of Medicine and Pediatrics, Institute for Human Genetics, University of California San Francisco, San Francisco, California, United States of America

We like to think that our genome is rock-solid, that it is dependable, there for us when we need it. The truth is far from that. By fits and starts, our species' collective genome is undulating, reshaping itself with eruptions of genomic lava and clashes of sequence tectonics, at once both marvelous and unsettling. We are unaware of this tumult within us until we are confronted with disease in ourselves, our friends, or our family.

**Image 1:**
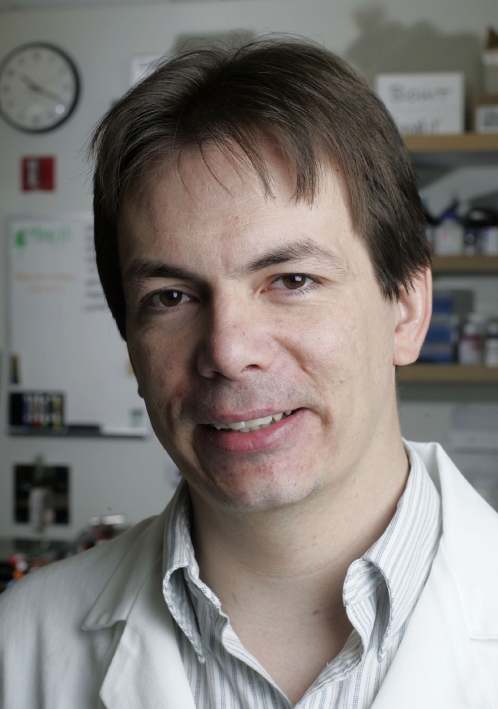
Evan Eichler (Image: Ron Wurzer/AP)

Evan Eichler is a man obsessed with this process, and to speak with him is a study in contrasts (Image 1). An unassuming Canadian, Eichler is a student of genomic architecture, the arrangement of sequences in our genome, and their evolution. Eichler grew up on a farm in Manitoba, married his college sweetheart, and now lives together with her and their four children in the mountains east of Seattle. As we walked up the hill to my office during his recent visit to UCSF, he talked about being an early riser, taking his son to band practice before school, and then driving the 30 miles to work in his Toyota. Eichler is a man bristling with excitement for his discoveries, but holding it in check by a tradition of modesty. He has consistently followed his own path, chosen career opportunities that were dictated not by politics or peer pressure but rather by what feels like a good fit for him.

Our conversation ranged from tiny triplet repeats to large and complicated duplications, some of which harbor genes of uniquely human import, and the process of their discoveries.

**Gitschier:** Your thesis advisor, David Nelson, told me that when you came to Baylor [College of Medicine] as a first-year graduate student, you already knew you wanted to study genome evolution.

**Eichler:** That's true.

**Gitschier:** How did you know that?

**Eichler:** I was at the University of Saskatchewan [as an undergraduate] and I was in Biology. They didn't have a genetics program, but I knew even before I went there that I wanted to do genetics.

**Gitschier:** And how did you know *that*?

**Eichler:** It started out in grade 9 or 10. My family grew up on a farm, and we started to raise angora rabbits for the purpose of their wool. My mother was one of those folks for whom everything had to be done naturally. So we had to pull the wool, we couldn't clip the wool. (It's OK—the rabbits are fine with it!) She spun her own wool. And she didn't believe in dying the wool.

She said to me, “I want different colors of wool, but I don't want to use dyes.” And in grade 9, I learned how to use the Punnett Square to keep track of the five gene coat color system in rabbits. I got a little textbook, and I started breeding these rabbits. I joke that that was the only time I ever did classical genetics!

I did those experiments, and within a couple of generations, I got all the colors that she wanted. I could breed them true. My mother was so impressed! It's amazing what you can do!

At that point, I decided that genetics was what I wanted to do. And by the high school years, after reading stuff, I realized I wanted to do human genetics.

So my father looked into a number of different universities to check out the genetics programs, but I ended up settling on a place where there was no genetics, because it was close to home.

I wanted to take more molecular courses, but I ended up taking more ecology, evolution, and anthropology, because it was part of the curriculum. They [the faculty there] didn't believe in what they called “reductionist” biology.

**Gitschier:** But that served you so well in the long run!

**Eichler:** This is, I think, where my interest in evolution [was] sparked. And when I finished my Bachelor's degree, one of my professors said, “If you want to do human genetics, you have to get an MD”.

So I thought about that, and I took a year off. I got a fellowship to study in Munich, at a veterinary research institute. And there I got real exposure to research, and that's where I applied to different [graduate] schools—Sick Children's in Toronto, Hopkins, Yale, and Baylor. And got invitations from those schools but eventually went to Baylor. I thought the research there was comparable to that at the big names, and I thought the people like David and Phil Soriano, who had interviewed me on the phone, had this folksy feel—really down to earth, but very high energy and, obviously, top quality.

My uncles gave me a hard time: “You had a chance to go to an Ivy League school and you're going to *Texas*!” But at that time, Tom Caskey had such a great enterprise there. He had such great taste and recruited such impressive faculty. I was so happy there.

**Gitschier:** And you went, without even looking at it?

**Eichler:** I hadn't seen the city, and if I *had*, maybe I would have changed my mind! I went there. I wasn't married yet, so I had to fly back [to Canada], marry my wife in the middle of midterms and bring her down, and she said “I cannot believe you have moved me from Canada to *here*.” She hated it for the first 6 months, but eventually she grew to like the city and the Medical Center.

I was extremely lucky to find David Nelson as my mentor. There was an instantaneous click—a chemistry. He gave me complete freedom, but he was an academic rock. When I came up with ideas, he would quickly find where the flaws were and then allow me to go on and pursue them. I was one of those strange students who actually wrote two qualifying exams because I couldn't decide what I wanted to do. My committee told me to focus, but David said, “Eh, do what you want,” shrugging his shoulders.

**Gitschier:** I can just hear him saying that!

**Eichler:** And I loved this whole Fragile X thing—the idea of a mutation being dynamic, and a premutation state. The anticipation phenomenon [that disease risk increases in subsequent generations, now known to be due to triplet repeat expansion] had been rejected by a lot of mainstream geneticists ten years before—they thought it was just ascertainment bias. And then to have it all resolved by Ying-Hui Fu and David, to be there at that moment when those *Cell* papers were coming out! When I came to that lab, from early on, I was interested in studying that process from an evolutionary perspective.

When I think about duplications, I think about them *exactly* along those same lines—as a dynamic mutational process. Instead of slippage of triplet repeats, it is non-allelic homologous recombination. These regions, unlike most of the genome, break all the rules. They can have very accelerated rates, and then pause, if there is selection, either positive or negative. They beat to their own tempo.

I started working on the mechanism of the instability. Why do triplet repeats expand at all? From Ying-Hui's sequencing work, we knew there were AGG interruptions in the CGG repeats. So working with David and Steve Warren, we came up with a model that a loss of AGG interruptions would predispose alleles to change. We showed that alleles that lacked the AGG interruptions moved toward premutation and disease state much more quickly within the human population. Some populations, such as Tunisian Jews, had a disproportionately large number of uninterrupted alleles, and in these same populations, Fragile X syndrome was much higher. So what mattered [in promoting instability] was a pure tract of CGG rather than the total number of repeats.

**Gitschier:** Other primates don't have fragile sites, do they?

**Eichler:** Not that we ever have observed, and they also have many more interruptions in the CGG sequences, and different types of interruptions in different species. And you never see the amplification and the fragile site.

All microsatellite lengths in other species are shorter on average than in humans. Even the polyglutamine-coding tracts. It's almost as if the human species has been sloppy to allow these types of track lengths to increase, unless they have some kind of benefit.

At the end of my PhD, I got a side project going—to map the Emery muscular dystrophy gene. So I started mapping cosmid clones in the Xq28 region, and, lo and behold, as I was walking across that region I got some unusual results—cosmids that should have come from the X-chromosome, but hybridized by FISH to multiple locations. One clone had the creatine transporter locus, and it hybridized clearly to both Xq28 and 16p11.2. And another one—the adrenal leukodystrophy locus—it hybridized to four locations in addition to Xq28.

That's when I started the idea of looking at duplications and copy number variation, in 1996, 1997.

**Gitschier:** At the time, were you really thinking about copy number variation *within* species, or just about the evolution of segmental duplications?

**Eichler:** At that time I would have been thinking about segmental duplications and copy number variations *between* species. But it was shortly thereafter—1997, 1998—that copy number variations *within* a species became apparent.

There were pericentromeric duplications, proximal to the Pradi-Willi region on 15q, published by Marc Lalande, and another paper by John Barber reporting larger 16p11 copy-number variations. Both of these papers showed copy number variation in large segmental duplications in normal individuals, and most of this variation was thought to be evolutionarily quite young, less than 10,000 years. Barb Trask had shown in 1998 that the subtelomeric regions had dramatic structural variation between species and within humans. And all of this was *way* preceding any of the hype in 2004 about copy number variation.

**Gitschier:** I know what you mean. Suddenly there appeared an acronym “CNV” [copy number variation] for something that had been known about for quite a while.

**Eichler:** One of the problems with genomics, in particular, is that the collective memory seems to be about five minutes to twelve [o'clock]. It's where people are at the last three or four minutes before the bell tolls that seems to matter and they forget everything that went before. It's a little bit frustrating, but I imagine everybody feels this at some point. It's not that these ideas appear out of nowhere. It's not a vacuum and suddenly a light goes on one day! They're built upon many studies over many years.

So between 1996 and 1998, we were already thinking about copy number variation, but we wanted first to understand the organization [of duplications] in humans, and then understand the difference between humans and other primates, and then focus again on humans, distinguishing normal and disease-causing variation.

**Gitschier:** You must have great computational skills to do this kind of work.

**Eichler:** I'm not a programmer, and I never took a single class in it. But David was a big fan of UNIX. I never was afraid of moving big data sets around.

What happened to me was that we had done these anecdotal studies looking at duplications, repeats, variations between species, and then a couple of things happened.

First, I took my faculty position at Case Western Reserve University. Hunt [Willard] and Aravinda [Chakravarti] recruited me. David and I had been working on a paper with Aravinda on Fragile X haplotype analysis. He heard I was job hunting and said, “Why don't you come and look over here?” I thought, “That is a great place!” Hunt had the chromosome structure part, Aravinda was doing the human genetics disease angle, Rob Nicholls doing the Pradi-Willi/Angelman work. It felt like a natural fit with that whole faculty. And Cleveland is great! It was cheap. The people there seemed so down to earth. It was just a perfect fit for me and my research. So I moved there in 1997.

**Gitschier:** So now, let's talk about how you moved to the whole genome problem, which I assume is the second “thing” that happened.

**Eichler:** Yes, moving from studying individuals' genes, duplications, and variations between species, to genome-wide. This was right at the time when the genome project was basically hoping to finish up in the next few years.

There was a culture shift, in 1998, as follows: The whole [publicly sponsored] genome project had been done, up to that point, methodically, slowly, BAC by BAC, fosmid by fosmid, cosmid by cosmid, people assigned chromosomes and doing their regions, reporting their results at chromosome-specific workshops. And at that point, there was a shift, essentially: [Craig] Venter. Venter saying he was going to do it faster and better and sell it as a marketable product.

So [as part of this race] I was brought to NIH—first time I had ever been there. I knew that they [segmental duplications] would be difficult [to identify], and I knew they would be important. We had done some basic analysis to see how good this working draft sequence would be as opposed to finished sequence.

I remember saying that a working draft [as opposed to a careful orderly description] would mess up duplications completely, and that we wouldn't resolve them well and it would be a disaster for my research, blah blah.

But I could tell right then that it didn't matter. They were going to sequence *lots* of clones, with sequences deposited into GenBank in the next 13–15 months. MIT at that point picked up a lot of the sequencing capacity, and Wash U [Washington University] was committed to finished, high-quality sequencing, most in ordered maps, but not all of them were.

So after that, I got a call from Eric Lander. He said, “Evan, we're going to have all these assemblies of the human genome soon, we'd sure like it if you were willing to do a genome-wide analysis [to help determine regions of duplication]. Have you ever done a genome-wide analysis?”

And I said, “No.”

He said, “Well, *can* you do it, and can you do it in 4–5 weeks?” This was around 2000.

I lied. I said, “We can do this.”

I knew we could do it, but I didn't know we could do it that quickly! And so we went ahead. The sequence came in. We had to come up with a pipeline to analyze duplications within the assembly. I had an *awesome* student, Jeff Bailey, who was better at computation than he was at the bench. So we sat down and drafted what we would need—how we would do it: remove the repeats, line up the sequences, genome by genome, we'd clip—there was a heuristic involved.

And then we had to execute it, but we didn't have enough computers. There was no cluster or super-computer that we had access to.

So I walked over to Hunt's office and said, “Hunt, I have an opportunity and I need some machines. And I have a guy who is actually capable of re-writing the operating system and putting Linux on all these.” So he said, “OK here's $13,000, see what you can do.”

**Gitschier:** That's not very much.

**Eichler:** No! But we went out and literally bought off-the-shelf from Computer City a whole bunch of machines—I think they were Dells—and we strung them up on my lab bench—there were 15 of them. I have a picture of it somewhere. And Jeff wrote some script that would distribute the load across the machines so we could parallelize the operation.

It was the middle of summer in Cleveland. And things would go for a week and crash. And the process was such that you'd have to start all over again. Two weeks in—crash.

**Gitschier:** Power outages?

**Eichler:** No—heat. The rooms weren't air conditioned *enough* to deal with the heat that was generated from 15 computers strung together, side-by-side. We had a little maelstrom of heat. So the critical component for this first cluster that we built was a Kmart fan—actually three of them—that we stuck in the back and blew the heat away from the back of the chassis of the computers, and it finally ran to the end. We ended up a little bit late, two weeks late I think, but we did our first genome-wide analysis.

And it was *really* disappointing. We realized that the first assemblies had screwed up big time—something like 20% of the genome was in these blocks of duplication, and 90% were false positives. We were bummed out because we had put all this energy into a duplication map. We had all these ideas for evolution and disease, and we realized that we didn't have it yet!

So we reported this back, and they were like—OK, we've got to fix this. So we gave all the coordinates to [David] Haussler [developer of the UCSC genome browser]. And additional assemblies went on to be more rigorous.

But to us, it wasn't what we wanted. Which were the false positives, and which were the real duplications?

So what we did—and here is where we got into a little bit of trouble—I knew Venter was doing his genome assembly a *different* way, and I knew that his assembly method would *miss* the duplications *completely*, because they couldn't actually assemble within a duplicated region. There would be “mate-pair” violations [mismatches of two shot-gun end-sequences], and they would just throw out the discrepant reads.

So we came up with this idea, which is fairly simplistic. We knew that the best part of the public [genome sequencing effort] was that they had individual haplotype BACs—150,000 base pairs in individual clones—that were good for orderly assembly. And, we knew that the best part of Craig's was this whole genome shot-gun approach. So if we could take the raw data from both projects and merge them—we'll use the depth of coverage [from Craig's shot-gun sequences] as a dipstick for duplication, and we'll take every BAC [from the public genome project]—all 36,000 of them—and we'll align all of Craig's reads against them. Wherever we see excess depth of coverage and wherever we see excess divergence will indicate a potential duplication.

So we tested it, and we had a 95% hit rate on our duplications. We could detect duplications that were big. By 2002, before any [final genome] assembly came out, we had a duplication map, and that was published [in *Science*] as a map for future studies.

And that's where I got into problems, because I was analyzing both public and private human genomes prior to either being published.

**Gitschier:** So you had bought into the Celera database?

**Eichler:** I didn't. I collaborated—I collaborated with the public and I collaborated with Venter at the same time.

**Gitschier:** Were there others who did that, too?

**Eichler:** I don't think so, because I remember getting phone calls warning me to be careful!

I was interested only in the scientific question, not the politics. And I explained that to a number of people including Francis [Collins] and Eric [Lander], and Mark Adams at Celera, with whom I had an established collaboration. I had to keep a wall of China up between the two sources. People eventually understood that I wasn't contaminating the well, on either side.

There were two things working in my favor—most of all I was naïve, and I just didn't understand a lot of things that had gone on with Congress, and things that had gone on trying to stifle one project versus the other. The second thing going for me was that I was blinded by getting the duplications sorted out. This would be the greatest thing since sliced bread in my life.

**Gitschier:** And it was!

**Eichler:** It was great, and fun. For us, that was a watershed moment. We used that information to predict regions of rapid evolutionary turnover as well as regions that we believed were disease hotspots, like the autism locus at 16p11.2. And we decided to systematically go in and look for structural variation in these regions.

**Gitschier:** Tell me more about the duplications.

**Eichler:** If you try to reconstruct the whole evolutionary history of the duplications themselves, what you notice is a couple of things. One is that humans have too many interspersed duplications compared to other mammals.

Point number two: the duplication architecture is very complex, suggesting that there has been a series of events creating almost every duplication block, of which there are about 400 in the human genome. Most of these have been created over a period of 10–15 million years, where it seems that most of the activity was around the time of the common ancestor to human and chimp and gorilla.

And then here's the kicker: if you reconstruct the entire history of these, they provide a framework. Because duplications swap material between them, they share evolutionary history. You can build a tree of relationships of the segmental duplications.

What you see is that most of the expansions have occurred on about half a dozen human chromosomes, and most of these expansions lead to these architectures, such as on Chromosomes 16 and 17, where you now have *big* blocks of duplication flanking a region and sensitizing it to microdeletion and microduplication.

So, you ask yourself, “Why these big blocks?”

Here's what I think, although we haven't definitively proven it yet. If you look at the centers of these big blocks of duplications—what we call the cores—these tend to carry rapidly evolving genes embedded within them.

So we're coming to a new and what I think is an important paradigm here: Architecture that is predisposing to microduplication and microdeletion is there at the benefit of having these newly minted genes propagating and expanding across the human genome.

There are now half a dozen gene families that have been published—some by us, some by others—which show these signatures, and most of the duplication architecture seems to be almost a genetic hitchhiker—these cores that have landed in new areas, picked up flanking material and duplicated again to other sites.

I would have to think that solving this riddle of what the function of those genes are would be tremendous. The genes that are in there tend to mark the oldest and the deepest part of the duplication block. The block itself is a mosaic of different pieces. There are bits of pieces of genes, some are transcribed, some are not, most are neutrally evolving. But the cores carry genes that show strong signatures of positive selection. These are genes that are smack in the middle of hundreds of kb [kilobases] of complex duplication territory, where Affymetrix, Agilent, and the SNP people have feared to tread! This is the “un-HapMap-able” region of the human genome. And if there were any association with any disease, people would have missed it because there is no type of genotyping technology to actually assay.

And here's the rub—these genes are not only embedded in complex duplications, they are even copy number variant between humans. We have a gene family we call “Morpheus”, which we published in 2001. Some people have 20 copies, some have 16 copies.

So, what are the functions of these genes? Tough question! This is the geneticist's worst nightmare: Mice don't have them, so you can't knock them out. There are multiple copies in humans, so would be tough to genotype. They are too far away from any flanking tagged SNP to find any type of association. So there are a whole series of black holes.

**Gitschier:** What has been the thing about your research that had you the most jazzed?

**Eichler:** I'm still jazzed!

Four or five years ago I made a conscious decision to go back to my roots—to go back to human disease. Up to that point, I was focusing on human duplications strictly from the perspective of structural variation in humans and variation among primates. So taking what I learnt from David Nelson and Jim Lupski, I've come full circle, because we are now studying children with disease, and we're finding what we think are causes—at least associations—now. But we're doing it from the perspective of looking at the genomic architecture, as opposed to linkage or association.

Both of those make me feel good—I love the evolutionary history of the human genome, and I'm completely unapologetic that I am anthropocentric—that if I were doing the same thing in *Drosophilia*, it would not interest me. But in humans—I care how we tick! That we can walk around with all of this *stuff*. It's almost liberating—the fact that there is no perfect genome—that all of us are made up of deletions and structural changes and copy number variations.

It's amazing that any of us are “normal”. And maybe none of us really are—and that's the beauty of it!

